# Refactoring the *λ* phage lytic/lysogenic decision with a synthetic regulator

**DOI:** 10.1002/mbo3.352

**Published:** 2016-03-14

**Authors:** Gonzalo Durante‐Rodríguez, José Miguel Mancheño, Eduardo Díaz, Manuel Carmona

**Affiliations:** ^1^ Environmental Biology DepartmentCentro de Investigaciones Biológicas‐CSICRamiro de Maeztu 928040MadridSpain; ^2^ Institute of Physical Chemistry Rocasolano‐CSICSerrano 11928006MadridSpain

**Keywords:** *Azoarcus*, protein engineering, synthetic biology, transcriptional regulation

## Abstract

In this work, we explore the refactoring of the circuitry of *λ* phage by engineering a new‐to‐nature regulator that responds to an ad hoc input signal that behaves orthogonal with respect to the host cell. We tailored a chimeric regulator, termed Q*λ*, between the CI protein of the *λ* phage and the BzdR repressor from *Azoarcus* sp. strain CIB that responds to benzoyl‐CoA. When the Q*λ* was expressed in the appropriate *Escherichia coli* cells, it was able to reprogram the lytic/lysogenic *λ* phage decision according to the intracellular production of benzoyl‐CoA. Our results are also an example of how generating new artificial regulators that respond to effectors of choice may be useful to control different cellular processes.

## Introduction

Gene switches and protein devices have wide utility in synthetic biology and multiple elements are needed to construct advanced circuitry or to control organism physiology. The *λ* phage lytic/lysogenic decision circuit represents a network that has been extensively used for circuit reprogramming (Vohradsky 2001; Atsumi and Little 2004; Little 2010). Most of the studies involved the two antagonistic repressors, CI and Cro (Astromoff and Ptashne 1995; Little 2010). The *λ*CI repressor encoded by the *cI* gene of *λ* phage represses the *P*_*L*_ and *P*_*R*_ promoter, and therefore the *λ* phage lytic cycle (Stayrook et al. 2008; Hochschild and Lewis 2009). The N‐terminal domain of CI (*λ*NCI; residues 1–92) mediates in DNA‐binding, and is connected by a 37‐residue linker to the C‐terminal domain (*λ*CCI; residues 93–101) that participates in the dimerization of the repressor and also in the interaction between dimers through a protease‐sensitive subdomain (Pabo and Lewis 1982; Astromoff and Ptashne 1995; Bell et al. 2000). The CI repressor is expressed from the *P*_*RM*_ promoter and binds cooperatively to two operators, *O*_*R*_1 and *O*_*R*_2, to repress the *P*_*R*_ lytic promoter and thus blocking transcription of the *cro* gene (Reichardt and Kaiser 1971; Hawley and McClure 1982; Michalowski et al. 2004). When *Escherichia coli* senses an environmental stress signal, such as cell and/or phage DNA damage, for example by ultraviolet irradiation, thymine starvation, or other environmental stress signals, the *λ* prophage is induced to the lytic cycle by the RecA‐mediated proteolytic cleavage of the CI repressor (Herskowitz 1973; Craig and Roberts 1981). Cro protein, that is essential for *λ* phage lytic development (Schubert et al. 2013), is expressed from *P*_*R*_ and prevents CI expression, presumably by virtue of its high affinity for the *O*_*R*_3 operator, where it can bind to repress *P*_*RM*_ by direct CI positive feedback.

The CI repressor can be ascribed to the HTH‐XRE‐family of proteins (SMART release, available on the Web Wide Web at http://smart.embl-heidelberg.de/smart/do_annotation.pl?BLAST=DUMMY&DOMAIN=HTH_XRE). The XRE (xenobiotic response element) family of transcriptional regulators is the second most extended family of regulators in bacteria with an helix‐turn‐helix (HTH) DNA‐binding motif similar to that of the well‐characterized Cro protein of *λ* phage (Roberts et al. 1977; Sauer et al. 1982; Barragán et al. 2005). Environmental signals that activate XRE‐family proteins are widely variable, and they range from small molecular effectors to large proteins. Although the XRE‐family regulators share a characteristic N‐terminal HTH DNA‐binding domain (Pfam01381), their C‐terminal effector‐binding region is highly variable. It is well known that the N‐terminal and linker domains of CI (*λ*NLCI) lack the motifs involved in dimerization (Di Lallo et al. 1999). Therefore, *λ*NLCI has been used as a genetic tool to study the ability of certain proteins, or protein domains, to dimerize after their fusion with the *λ*NLCI domain, by monitoring the repression of the target *P*_*R*_ promoter (Di Lallo et al. 1999). However, as far as we know, none of these chimeric CI regulators was tested for its ability to reprogram the lifestyle of the *λ* phage.

**Figure 1:**
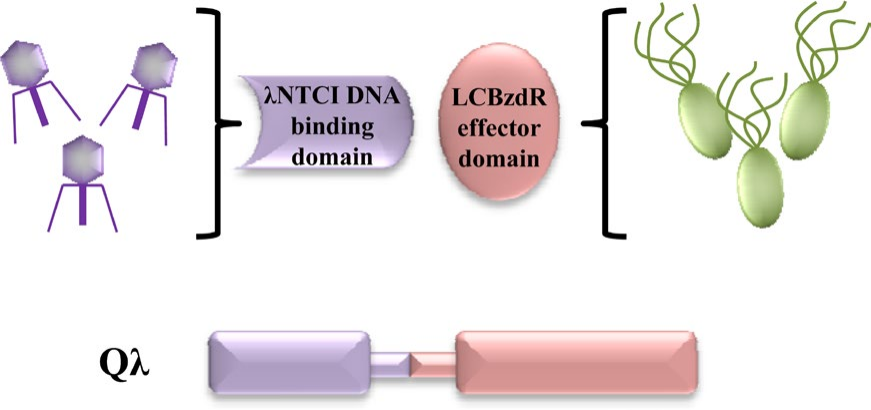
Rationale of the construction of the interkingdom Q*λ* chimera. Q*λ* is a chimera factored by fusion of two functional modules, a DNA‐binding domain from the CI repressor of *λ* phage (*λ* NLCI) and an effector‐recognition domain from the bacterial BzdR protein (LCBzdR). *λ* NLCI, N‐terminal and linker domains of CI.

In this work, we explore the refactoring of the circuitry of *λ* phage by engineering a new‐to‐nature regulator that responds to an ad hoc input signal that does not interfere with the normal metabolic network of the host cell, that is, it is to some extent orthogonal, and hence avoid unexpected changes in the behavior of the circuit. To design such regulatory protein, we have engineered a chimera between the CI protein and the BzdR protein from bacterium *Azoarcus* sp. strain CIB. BzdR is a transcriptional regulator involved in the control of the anaerobic catabolism of benzoate that recognizes benzoyl‐CoA as inducer molecule (Barragán et al. 2005). The multidomain organization of BzdR consists of an N‐terminal domain, harboring a HTH fold, connected through a linker sequence to a C‐terminal domain (LCBzdR; residues 91–298) that shows similarity to the *E. coli* shikimate kinase I (SKI) and recognizes the inducer molecule benzoyl‐CoA alleviating BzdR repression on the target promoter (Barragán et al. 2005; Durante‐Rodríguez et al. 2010, 2013). Here, we tailored an interkingdom chimeric regulator, termed Q*λ*, factored by fusion of two functional modules, a DNA‐binding domain from the CI repressor of *λ* phage and an effector‐recognition domain from the bacterial BzdR protein (Fig. 1). The Q*λ*, when expressed in the appropriate *E. coli* cells was able to reprogram the lytic/lysogenic *λ* phage decision according to the intracellular production of the orthogonal input signal benzoyl‐CoA. Our results are also an example of how generating artificial regulators that respond to selected input signals to control different cellular processes.

## Material and Methods

### Bacterial strains, bacteriophages, plasmids, and growth conditions

The *E. coli* strains, phages, as well as the plasmids used in this work, are listed in Table 1. *Escherichia coli* cells were grown at 37°C on lysogeny broth (LB) medium (Sambrook and Rusell 2001). Suspension Medium (SM) medium for bacteria and *λ* phage dilutions were prepared as described by Miller (1972). Where appropriate, antibiotics were added at the following concentrations: ampicillin, 100 *μ*g mL^−1^; kanamycin 50 *μ*g mL^−1^; chloramphenicol, 30 *μ*g mL^−1^.

**Table 1:** Bacterial strains, bacteriophages, and plasmids used in this work

Strain or plasmid	Relevant phenotype and/or genotype	Reference or source
*Escherichia coli* strains		
DH5*α*	*endA1 hsdR17 supE44 thi‐1 recA1 gyrA*(Nal^r^)	Sambrook and Rusell (2001)
	*relA1Δ(argF‐lac) U169 depR* ø*80dlacΔ(lacZ)M15*	
MV1190	Δ(*lac‐pro*), *thi*,* supE*, Δ(*srl‐recA*)*306*::Tn*10*, (F' *traD36*)	Bio‐Rad
	*lacI* ^*q*^ *ZΔM15*,* proAB*	
Bacteriophages		
*λ* phage	EMBL4	Frischauf et al. (1983)
Plasmids		
pCI‐CAT	Ap^r^, pC132 derivative harboring the *cat* gene of Tn*906* in‐frame with the 5′ portion of *λ*CI	Di Lallo et al. (1999)
pNLCI	Ap^r^, pCI‐CAT derivate that express the *λ*NLCI module	This work
pQ*λ*	Ap^r^, pNLCI derivative that express the Q*λ* chimera under control of the *lac* operator	This work
pCK01BzdA	Cm^r^, pCK01 derivative that express the *bzdA* gene under the control of the *Plac* promoter	Barragán et al. (2005)
pSJ6	Km^r^, broad‐host‐range plasmid that includes a 4.0 kb	Jaenecke et al. (1996)
	NotI cassette harboring the fusion *P*_*R*_ *:tnp'::'lacZ*	

Ap^r^, ampicillin resistant; Cm^r^, chloramphenicol resistant; Km^r^, kanamycin resistant.

### Molecular biology techniques

Recombinant DNA techniques were carried out by published methods (Sambrook and Rusell 2001). Plasmid DNA was prepared with High Pure plasmid isolation kit (Roche Applied Science, Penzberg, Germany). DNA fragments were purified with Gene‐Clean Turbo (Qbiogene, Inc., Carlsbad, CA, USA). Oligonucleotides were supplied by Sigma‐Aldrich (Carlsbad, CA, USA). All cloned inserts and DNA fragments were confirmed by DNA sequencing through an ABI Prism 377 automated DNA sequencer (Applied Biosystems Inc., Waltham, MA, USA). Transformation of *E. coli* cells was carried out by using the RbCl method (Sambrook and Rusell 2001) or by electroporation (Gene Pulser; Bio‐Rad, Hercules, CA, USA).

### Plasmids construction

Plasmid pNLCI expressing the *λ*NLCI protein was constructed by deleting the *cat* gene, encoding chloramphenicol acetyl transferase, from the pCI‐CAT plasmid (Di Lallo et al. 1999). To this end, plasmid pCI‐CAT was HincII/SmaI double digested and further religated. Plasmid pQ*λ* was constructed by cloning into HincII/BamHI double‐digested pNLCI plasmid a 642‐bp StuI/BamHI *LCBzdR* gene fragment that codes for the linker and the C‐terminal domain of BzdR. *LCBzdR* was PCR‐amplified from the *Azoarcus* sp. CIB genome by using oligonucleotides 3‐newlynk (5′‐GAAGGCCTGCGCGAGGAGGCGGAGCAGTC‐3′; an engineered StuI site is underlined) and 5‐C2 (5′‐CGGGATCCTCAGCGTGCCAGGACTTCGAGG‐3′; an engineered BamHI site is underlined).

### Enzymatic assays

*β*‐galactosidase activities were measured using permeabilized cells as described by Miller (1972).

### Bacteriophage infection assays

We tested the immunity to *λ* infection as described by Di Lallo et al. (1999). Bacterial strains were grown in LB to about 1 × 10^8^ cells mL^−1^, centrifuged and resuspended in SM supplemented with 10 mmol/L MgSO_4_ and 5 mmol/L CaCl_2_, and poured with soft agar on LB plates. Spots of several dilutions of *λ* stock phage were then added, the plates were incubated for 18 h at 37°C, and the number of lysis plaques was determined.

## Results and Discussion

### Engineering a new‐to‐nature Q*λ* regulator

**Figure 2:**
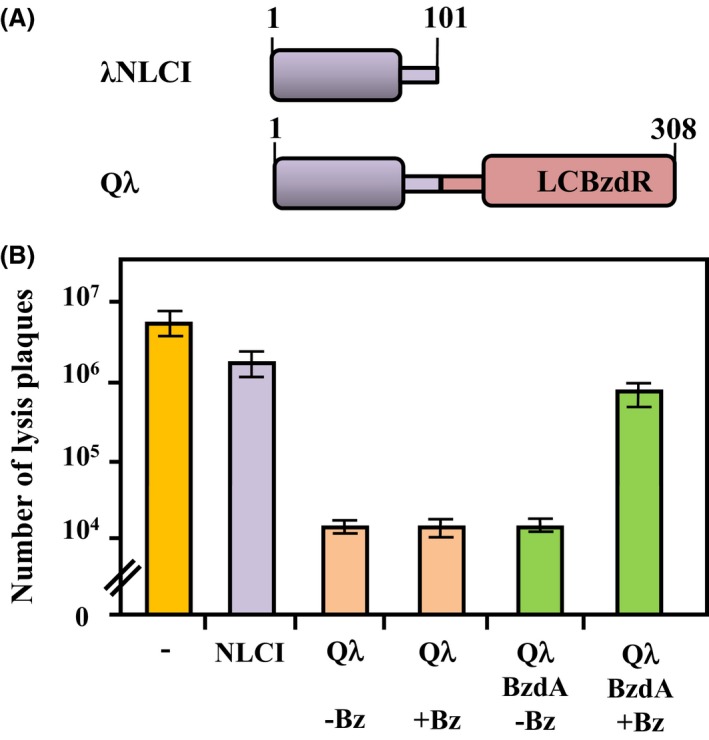
Modular architecture and functionality of the Q*λ* chimera. (A) Scheme of the modular architecture of the *λ* NLCI protein and Q*λ* chimera. The N‐terminal domain and linker region of CI (residues 1–101 from CI protein) are shown in violet. The C‐terminal domain and linker region of BzdR (residues 91–298 from BzdR protein) are shown in red. (B) *Escherichia coli* MV1190 parental strain (−) or MV1190 strain carrying plasmids pNLCI, expresess *λ* NLCI (NLCI), pQ *λ*, expresess Q*λ* (Q*λ*), and pQ *λ* plus pCK01BzdA, expresses BzdA (BzdA), were grown in LB medium in the absence (−Bz) or presence (+Bz) of 3 mmol/L benzoate, and then infected with *λ* phage as detailed in Material and Methods. Bars represent the average number of lysis plaques observed from three independent infection experiments ±SD. *λ* NLCI, N‐terminal and linker domains of CI.

With the aim of repurpose an existing regulatory module to make it responsive to metabolic effectors of choice and refactoring the *λ* lytic/lysogenic decision circuit, we accomplished the construction of a CI‐derived chimera, termed Q*λ*, made of two functional modules, that is, *λ*NLCI for binding to the *P*_*R*_ promoter and LCBzdR for binding to the effector signal (Fig. 2A). As indicated in the Introduction, BzdR is the transcriptional regulator that controls the *P*_*N*_ promoter responsible for the anaerobic catabolism of benzoate in *Azoarcus* sp. CIB. LCBzdR consists of the C‐terminal domain of the BzdR repressor that recognizes specifically benzoyl‐CoA as its effector molecule, and the linker region that was shown to be essential to transfer the conformational changes induced by benzoyl‐CoA to the N‐terminal DNA‐binding domain, thus leading to the release of the repressor from the *P*_*N*_ target promoter (Durante‐Rodríguez et al. 2010, 2013). Accordingly, a functional Q*λ* chimera might be able to repress the *λ P*_*R*_ promoter in the absence of benzoyl‐CoA, but should be able to de‐repress this promoter in the presence of benzoyl‐CoA. Thus, the rationale of our strategy was to express a functional Q*λ* chimera in a recombinant *E. coli* strain able to synthesize benzoyl‐CoA, and then confirm that after *λ* infection, the lytic cycle was responding to the new artificial input signal, benzoyl‐CoA. Moreover, since benzoyl‐CoA is not present in natural *E. coli* strains, it has a minimal impact in the general metabolism of the host cell which, in turn, should not interfere with the effector of the new regulatory circuit, that is, benzoyl‐CoA should behave to some extent orthogonal in respect to the host cell.

The *λ*NLCI protein and the Q*λ* chimera were constructed and expressed under control of the *Plac* promoter in plasmids pNLCI and pQ*λ* (Table 1), respectively, as indicated in Material and Methods. To validate the functionality of these proteins, we accomplished an immunity test to *λ* infection. To this end, we infected the parental *E. coli* MV1190 strain with the *λ* phage, obtaining an average of 6.4 × 10^6^ lysis plaques (Fig. 2B). When the recipient bacteria contained plasmid pNLCI expressing the *λ*NLCI protein, no significant variation in the number of the phage plaques was observed (Fig. 2B), indicating that, as expected, *λ*NLCI behaved as a nonfunctional CI repressor. However, when the recipient cells expressed the Q*λ* chimera, a decrease in the number of lysis plaques of about three orders of magnitude was observed (Fig. 2B). This result indicates that the Q*λ* chimera confers immunity to *λ* infection and therefore, suggests that this synthetic regulator is able to efficiently repress the lytic cycle of the *λ* phage. To check now whether the production of benzoyl‐CoA in the recipient cells could modulate the activity of the Q*λ* chimera and hence, the level of immunity to *λ* infection, we constructed an *E. coli* MV1190 strain harboring plasmid pCK01BzdA (Table 1) that expresses the *bzdA* gene (Barragán et al. 2005). *bzdA* encodes the benzoate‐CoA ligase that converts benzoate into benzoyl‐CoA as the first step in the anaerobic benzoate degradation pathway from *Azoarcus* sp. CIB, and it confers the ability to generate intracellular benzoyl‐CoA when cloned in *E. coli* cells grown in the presence of benzoate (López‐Barragán et al. 2004). Although the *E. coli* MV1190 cells expressing the Q*λ* and BzdA proteins were significantly immune to *λ* infection when grown in the absence of benzoate, they increased significantly their susceptibility to the lytic effect of *λ* phage when grown in the presence of benzoate (Fig. 2B). As expected, in the absence of the BzdA protein, benzoate does not cause any effect on the *E. coli* immunity to *λ* phage (Fig. 2B). Thus, these results suggest that the Q*λ* chimera is able to recognize benzoyl‐CoA as effector molecule, leading to the alleviation of the repression on the *P*_*R*_ promoter that controls the lytic cycle of *λ* phage. In summary, all these results have validated that the new‐to‐nature Q*λ* regulator represents a successful strategy to reprogram the life cycle of *λ* phage.

### The benzoyl‐CoA‐dependent Q*λ*/*P*_*R*_ regulatory system

**Figure 3:**
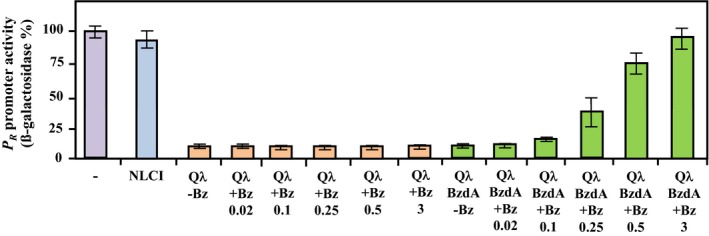
The Q*λ* chimera controls the *P*_*R*_ promoter in a benzoyl‐CoA‐dependent manner. *Escherichia coli* MV1190 cells carrying plasmid pSJ6, expresses *P*_*R*_ *::lacZ*, (−), or MV1190 cells carrying plasmid pSJ6 and plasmids pNLCI (NLCI), pQ *λ* (Q*λ*), or pQ *λ* plus pCK01BzdA (BzdA), were grown in LB medium in the absence (−Bz) or presence (+Bz) of 0.02, 0.1, 0.25, 0.5, or 3 mmol/L benzoate. *β*‐galactosidase activities were measured as described in Material and Methods, and they are represented as a percentage of the activity from *E. coli* MV1190 (pSJ6) cells (400 Miller units). Graphed values are the average from three independent experiments ±SD. NLCI, N‐terminal, and linker domains of CI.

The tight repression of the *P*_*R*_ promoter of *λ* phage by the CI repressor and its activation in the presence of CI inducers is a very useful trait that has been extensively used to develop conditional gene expression systems that respond to different environmental signals (Meyer et al. 1980). However, most of the CI/*P*_*R*_ derived regulatory couples that respond to a chemical signal rely on the transcriptional regulation of the *cI* gene, or on the native regulatory cascade that leads to the inactivation of the CI repressor. Therefore, it becomes interesting to explore new chemical signals that could be used to modulate directly the activity of the CI regulator within the target host cell. Since benzoyl‐CoA appears to be a metabolite that is not present in most microorganisms, it becomes a nice input signal to develop conditional expression systems that do not interfere, that is, orthogonal, with the general metabolism of the host cell. The results presented above suggested that the Q*λ* chimera was able to repress the *P*_*R*_ promoter involved in the lytic/lysogenic decision of the *λ* phage, and that the presence of benzoyl‐CoA within the cell alleviated such repression mechanism. To confirm the control of the *P*_*R*_ promoter by the Q*λ* repressor and the benzoyl‐CoA inducer, we monitored the expression of a *P*_*R*_ *::lacZ* reporter fusion. To this end, *E. coli* MV1190 strain harboring plasmid pSJ6, which expresses the *P*_*R*_ *::lacZ* fusion (Table 1), was transformed with plasmids pNLCI or pQ*λ* (Table 1), and the *β*‐galactosidase production was monitored. Figure 3 shows that cells expressing the *λ*NTCI protein have a *P*_*R*_ promoter activity (around 400 Miller units) similar to that of control cells lacking the CI repressor, revealing that, as expected, the *λ*NTCI protein is unable to repress the *P*_*R*_ promoter. On the contrary, cells expressing the Q*λ* chimera exhibited a 10‐fold decrease in the *β*‐galactosidase activity (Fig. 3), indicating that Q*λ* was repressing efficiently the *P*_*R*_ promoter. When the *E. coli* cells expressing the Q*λ* chimera and the BzdA ligase were grown in the absence of benzoate, the *P*_*R*_ promoter activity was inhibited (Fig. 3), but a significant activation of *P*_*R*_ was observed when increasing concentrations of benzoate was added to the culture medium, reaching *β*‐galactosidase levels similar to those of the control cells lacking CI at benzoate concentration above 0.5 mmol/L (Fig. 3).

**Figure 4:**
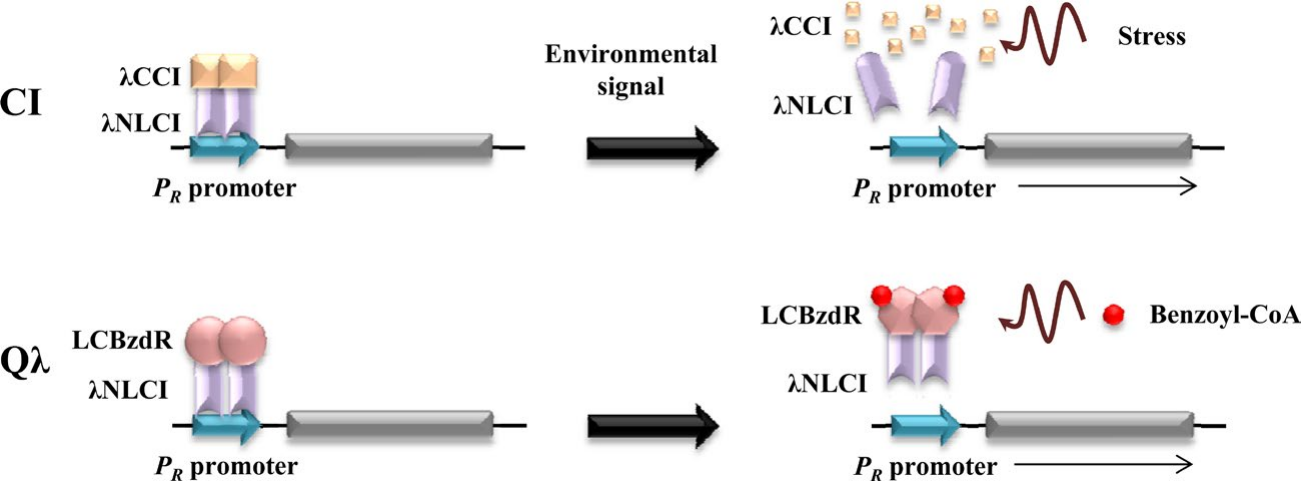
Model describing the Q*λ* chimera as a functional device. Comparison of the native and the artificial control of the *λ* *P*_*R*_ promoter by the CI repressor and the Q*λ* chimera, respectively. The CI repressor binds and represses the *P*_*R*_ promoter, and it releases from the DNA after certain environmental stress signals, such as UV radiation, leading to the de‐repression of *P*_*R*_. The Q*λ* chimera is also able to repress the *P*_*R*_ promoter, but de‐repression is triggered by the presence of benzoyl‐CoA, an inducer molecule that behaves orthogonal with respect to the host cell.

Analytical centrifugation and electron microscopy studies demonstrated that BzdR is a dimeric protein in solution (Durante‐Rodríguez et al. 2010), but C‐BzdR behaves as a globular monomer (Durante‐Rodríguez et al. 2013). Moreover, we have shown previously that chimeras that include the linker region from BzdR fused to monomeric domains or complete proteins (e.g., shikimate kinase) render functional dimeric proteins (Durante‐Rodríguez et al. 2013). Since we have demonstrated here that Q*λ* is able to repress the activity of the *P*_*R*_ promoter (Fig. 3), and it is known that *λ*NLCI lacks the motifs involved in dimerization and therefore, does not bind to *P*_*R*_ (Di Lallo et al. 1999), the linker sequence from LCBzR might be involved in the generation of a functional dimeric Q*λ* chimera. It was suggested that the LCBzdR domain has a LID‐like region that suffers a significant conformational change in the presence of the benzoyl‐CoA inducer molecule, which would be then transmitted to the NBzdR domain through the linker region. This benzoyl‐CoA‐induced conformational change triggers a structural modification at the DNA‐binding surfaces of the protein without affecting its dimeric state, which would end with the release of the regulator from the target promoter (Durante‐Rodríguez et al. 2010); a similar mechanism of action for the Q*λ* chimera controlling the *P*_*R*_ promoter can be predicted (Fig. 4).

All these results allowed us to conclude that the Q*λ*/*P*_*R*_ regulatory couple becomes a new artificial regulatory system that responds to an unusual inducer molecule, benzoyl‐CoA, and it constitutes an interesting alternative to current conditional gene expression systems based on the CI repressor and the *P*_*R*_ promoter.

## Conclusions

**Figure 5:**
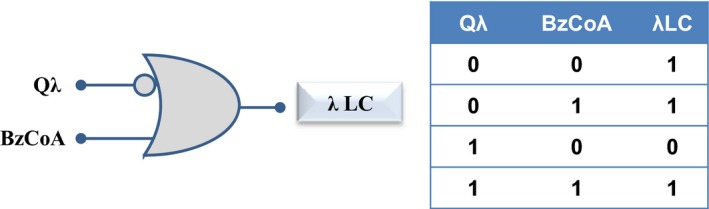
Logic gate and layout of Q*λ* device activity. At the left is presented the standard graphic annotation of the ORN gate representing the Q*λ* device activity. At the right, is shown the associated truth table of the ORN gate. BzCoA, benzoyl‐CoA; *λ* LC,* λ* lytic cycle.

This work shows that the interkingdom chimera Q*λ* is a functional protein that retains the activities predicted for its two constituent domains, namely recognition and binding to the target *P*_*R*_ promoter by the N‐terminal *λ*NLCI domain, and to the inducer molecule benzoyl‐CoA by the C‐terminal LCBzdR domain (Fig. 4). The Q*λ* chimera was used to refactor the lytic/lysogenic decision circuit of the *λ* phage using benzoyl‐CoA as the input signal of the engineered circuitry (Fig. 4). In this sense, Q*λ* device constitutes a molecular gate able to execute an ORN logic operation (Silva‐Rocha and de Lorenzo 2011). This ORN logic gate shows three elements, that is, the transcriptional regulator Q*λ*, the inducer benzoyl‐CoA and the target promoter *P*_*R*_, and the output is the induction of the lytic cycle except in the case of the presence of Q*λ* without the inducer benzoyl‐CoA (Fig. 5).

Modular experimental systems are needed to artificially control biological networks. As demonstrated here with the Q*λ* chimera, LCBzdR becomes a novel functional module that might be useful in the field of synthetic biology for engineering regulators *à la carte* that respond to benzoyl‐CoA, an inducer molecule that behaves orthogonal with respect to most microbial cells. Although this work has shown the value of the LCBzdR module by addressing the control of the *λ P*_*R*_ promoter, one can foresee the fusion of this module to the DNA‐binding domain of a wide variety of transcriptional regulators that control the activity of promoters driving the expression of genes involved in many different cellular processes. These novel devices designed *à la carte* would be useful to reprogram crucial cell functions, but also for engineering novel conditional gene expression systems of biotechnological interest that respond to benzoyl‐CoA as the effector molecule.

## Conflict of Interest

None declared.
